# *Cyclocarya paliurus* Reprograms the Flavonoid Biosynthesis Pathway Against Colletotrichum fructicola

**DOI:** 10.3389/fpls.2022.933484

**Published:** 2022-06-30

**Authors:** Xiang-Rong Zheng, Mao-Jiao Zhang, Yu-Hang Qiao, Ran Li, Noam Alkan, Jie-Yin Chen, Feng-Mao Chen

**Affiliations:** ^1^Collaborative Innovation Center of Sustainable Forestry in Southern China, College of Forestry, Nanjing Forestry University, Nanjing, China; ^2^State Key Laboratory for Biology of Plant Diseases and Insect Pests, Institute of Plant Protection, Chinese Academy of Agricultural Sciences, Beijing, China; ^3^Department of Postharvest Science, Agricultural Research Organization, Volcani Center, Rishon LeZion, Israel

**Keywords:** anthracnose, *Cyclocarya paliurus*, disease resistance, multiomics, flavonoid pathway

## Abstract

*Cyclocarya paliurus* is an endemic Chinese tree species with considerable medicinal, timber, and horticultural value. The anthracnose disease of *C. paliurus* is caused by the fungal pathogen *Colletotrichum fructicola*, which results in great losses in yield and quality. Here, resistance evaluation of six cultivars of *C. paliurus* exhibited varying degrees of resistance to *C. fructicola* infection, where Wufeng was the most resistant and Jinggangshan was the most susceptive. Physiological measurements and histochemical staining assays showed that the Wufeng cultivar exhibits intense reactive oxygen species accumulation and defense capabilities. A multiomics approach using RNA sequencing and metabolome analyses showed that resistance in *C. paliurus* (Wufeng) is related to early induction of reprogramming of the flavonoid biosynthesis pathway. *In vitro* antifungal assays revealed that the flavonoid extracts from resistant cultivars strongly inhibited *C. fructicola* hyphal growth than susceptible cultivars. Relative gene expression analysis further demonstrated the pivotal antifungal role of *C. paliurus* flavonoids in targeting *Colletotrichum* appressorium formation. Together, these results represent a novel resistance mechanism of *C. paliurus* against anthracnose through the reprogramming of flavonoids, which will lay a foundation for breeding anthracnose-resistant varieties and the application of flavonoid extraction of *C. paliurus* as a natural antifungal treatment.

## Introduction

*Cyclocarya paliurus* (Batal) Iljinskaja, the sole extant species in its genus, is an endemic Chinese tree species that is distributed in mountainous regions of subtropical China (Li et al., 2017; Kakar et al., 2020). The leaves are used as a nutraceutical tea that has been employed in traditional Chinese medicine to treat obesity and various illnesses for more than 1,000 years (Fang et al., 2011; Deng et al., 2015; Yang et al., 2018). In 1999, the *C. paliurus* tea from China became the first health tea approved by the Food and Drug Administration (FDA) (Wang and Wang, 2012; Kakar et al., 2020). In the past decade, significant attention has been devoted to *C. paliurus* for the antidiabetic, anti-inflammatory, antioxidant, and hepatoprotective properties of its leaf extracts, which are ascribed to synergies among its abundant secondary metabolites (Zhang et al., 2010; Fang et al., 2011; Xie et al., 2015, 2018; Liu et al., 2018a; Zhou et al., 2019). It is known that flavonoids are the main components of secondary metabolites in *C. paliurus*, which present broad physiological functions such as protecting the plant from environmental stresses (Zhang et al., 2021). *C. paliurus* flavonoids (CPFs) possess considerable health-promoting functions, with effects ranging from free radical scavenging to antibacterial and antihyperlipidemic activity (Li et al., 2011; Ma et al., 2015; Xie et al., 2015). Recent studies of CPFs are mainly concentrated on extraction methods, metabolite accumulation, and the improvement of human immunity (Zhang et al., 2010; Liu et al., 2018b; Xiong et al., 2018). However, to the best of our knowledge, the anti-phytopathogen or antifungal activity of CPFs in plants is still unclear.

The ascomycete genus *Colletotrichum* includes many species that infect more than 3,200 monocots and dicot plant species worldwide (Manire et al., 2002; O’Connell et al., 2012; Eloy et al., 2015). *Colletotrichum* is a hemibiotrophic fungus, which employs a short biotrophic lifestyle after penetration and quickly switches to a necrotrophic stage during its active pathogenic stage (O’Connell et al., 2012). Anthracnose caused by diverse *Colletotrichum* species was recently described as one of the most destructive diseases of *C. paliurus* to cause substantial losses in the yield, quality, and economic value, and *Colletotrichum fructicola* is the prevalent fungal pathogen responsible for *C. paliurus* anthracnose (Zhang et al., 2021). Although the chemical fungicides are an effective method to control anthracnose, their toxicity is always challenging to humans, animals, and the environment. Therefore, safe and long-term pathogen control strategies such as biofungicides and induced resistance should be explored (Zheng et al., 2021).

The hypersensitive response (HR) is a confined plant cell death against biotrophic pathogen ingression, which is characterized by the appearance of cell death (Balint-Kurti, 2019; Noman et al., 2020). From a molecular perspective, HR consists of serial events including reactive oxygen species (ROS) bursts, phytoalexin production, activation of mitogen-activated protein kinase (MAPK) cascades, calcium ion (Ca^2+^) influx, salicylic acid (SA) accumulation, and large-scale transcriptional reprogramming (Coll et al., 2011; Huysmans et al., 2017; Noman et al., 2020; Tian et al., 2020; Ren et al., 2021). HR formation and oxidative bursts are reactions associated with host resistance to *Colletotrichum* species at their biotrophic stage (Senthilkumar et al., 2012; Bhadauria et al., 2013). Transcriptome and physiological comparison of the resistant and susceptible tea cultivars that inoculation with *C. fructicola* showed that hydrogen peroxide (H_2_O_2_) accumulation and HR are critical mechanisms in tea resistance to anthracnose (Wang et al., 2018).

In this study, to elucidate the resistance mechanisms of *C. paliurus*, we evaluated the occurrence and severity of anthracnose on *C. paliurus* cultivars from six cultivars following *C. fructicola* infection. The most resistant cultivar from Wufeng and susceptible cultivar from Jinggangshan were selected for integrated analysis including physiological assays, RNA-seq, and metabolomics analyses to determine the mechanisms of *C. paliurus* resistance against *C. fructicola*, which can provide a basis for breeding research to enhance *C. paliurus* resistance.

## Results

### Evaluation of Anthracnose Resistance Among *Cyclocarya paliurus* Cultivars

**FIGURE 1:**
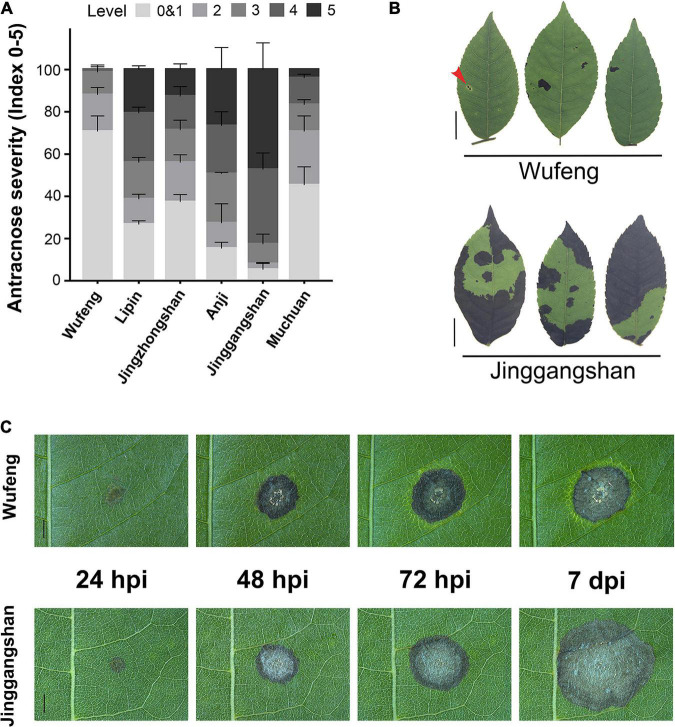
Comparative analysis of anthracnose on different cultivars of *Cyclocarya paliurus*. **(A)** Statistics of anthracnose susceptibility in six cultivars at 14 dpi, with disease symptoms, scored as described by Jiang et al. (2019). **(B)** Representative symptoms on Wufeng (above) and Jinggangshan (below) cultivars of *C. paliurus* after 14 dpi using intact plant inoculation. Leaf lesion surrounded by chlorosis indicated by a red arrow. Bars on left represent 1 cm. **(C)** Disease symptom development observed on Wufeng (above) and Jinggangshan (below) cultivar following detached leaf inoculation at 24, 48, and 72 h post-inoculation (hpi) and 7 dpi, respectively. Bars represent 2 mm.

The *C. paliurus* from six different cultivars were evaluated for anthracnose resistance following the detached leaf inoculation (DLI) and intact plant inoculation (IPI) methods. The disease indices and incidence rates differed significantly among the six cultivars at 14 days post-inoculation (dpi) (Supplementary Table 1). Among the IPI evaluation of six cultivars, the Wufeng is the most resistant cultivar to anthracnose that presents the lowest incidence rate and disease severity index, whereas the Jinggangshan was most susceptible to anthracnose (Figure 1A and Supplementary Table 1). Among samples from Jinggangshan, most lesions on infected leaves had enlarged and coalesced to form large necrotic areas, and in severe cases, the whole leaf had become withered and dropped by 14 dpi (Figure 1B). However, small restricted lesions were often surrounded by chlorosis in Wufeng samples by 14 dpi, which may have been related to disease resistance (Figure 1B). According to DLI, the initial symptoms began in both Wufeng and Jinggangshan samples as brown lesions appearing at the inoculation point at 24 h post-inoculation (hpi) (Figure 1C). Subsequently, brown spots spread as the disease progressed, causing typical symptoms of anthracnose, as acervuli formed and oozed gelatinous orange conidia masses. In Wufeng samples, leaf chlorosis began to form around the lesion at 72 hpi and did not expand further after it was entirely wrapped. The cultivars from Wufeng and Jinggangshan were therefore designated as resistant and susceptible for the subsequent analysis, respectively.

### The Resistant Cultivar of *Cyclocarya paliurus* Exhibits Intense Defense Responses

**FIGURE 2:**
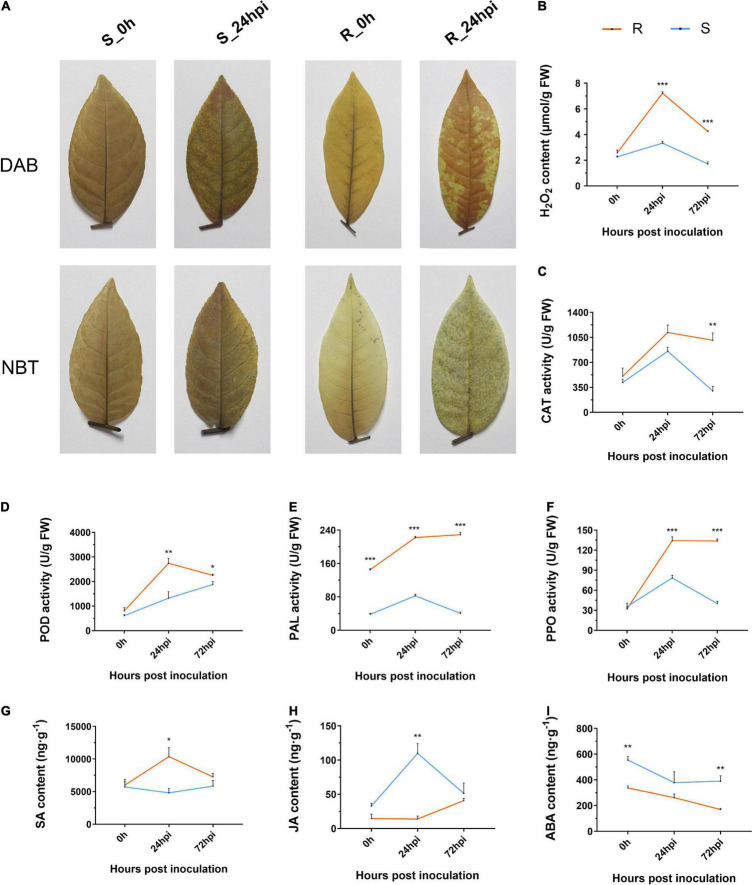
Changes in **(A)** histochemical staining and estimation of **(B)** H_2_O_2_ content, **(C)** CAT, **(D)** POD, **(E)** PAL, **(F)** PPO activities, **(G)** SA, **(H)** JA, and **(I)** ABA content of resistant and susceptible cultivars of *Cyclocarya paliurus* following *Colletotrichum fructicola* infection, respectively. R, resistant cultivar; S, susceptive cultivar. Bars represent the standard error (*n* = 3). *, **, and *** represent significant differences at *P* < 0.05, *P* < 0.01, and *P* < 0.001 (*t*-test), respectively.

Reactive oxygen species comprises both non-radical (H_2_O_2_) and free radical (O_2_⋅**^–^**) forms, which act as signaling molecules to control defense reactions (Hooijmaijers et al., 2012). In this study, we investigated the levels of ROS accumulation between resistance and susceptible cultivars of *C. paliurus via* the nitroblue tetrazolium chloride (NBT) and 3,3-dimethoxybenzidine (DAB) staining method. The staining patterns showed that the resistant cultivar had significantly higher levels of both O_2_⋅**^–^** and H_2_O_2_ accumulation at 24 hpi (Figure 2A). Quantification of the H_2_O_2_ content again showed that the resistance cultivar has a higher level of H_2_O_2_ than the susceptible cultivar when inoculated with the *C. fructicola* (Figure 2B). Moreover, we analyzed the enzyme activity of catalase (CAT) and peroxidase (POD) that are associated with ROS accumulation, both were consistently higher in the resistant cultivar than in the susceptible cultivar (Figures 2C,D). In addition, results showed that phenylalanine ammonia-lyase (PAL) and polyphenol oxidase (PPO) activities of resistant cultivar were significantly higher than that of susceptible cultivar at both 24 and 72 hpi (Figures 2E,F). Correspondingly, the SA content in leaves from the resistant cultivar was significantly higher than in those from the susceptible cultivar at 24 hpi (Figure 2G), while the opposite trend was observed for jasmonic acid (JA) concentration that generally antagonizes the level of SA (Figure 2H). In addition, the content of abscisic acid (ABA) displayed a similar downward tendency in both resistant and susceptible cultivars during pathogen infection (Figure 2I). Together, the resistant cultivar of *C. paliurus* initiates the significantly strong defense responses during infection, including ROS accumulation and hormone signaling.

### The Differential Expressed Genes of *Cyclocarya paliurus* Responses to *Colletotrichum fructicola*

To investigate the defense network of *C. paliurus* responses to *C. fructicola*, the resistant and susceptible cultivars of Wufeng and Jinggangshan were sampled at three time points (0, 24, and 72 hpi) and subjected to RNA-seq analysis. In total, 103.2 Gb clean reads were generated from 18 samples and a total of 336,157 transcripts were generated by *de novo* assembly (with an N50 of 1837 bp and N90 of 508 bp), and finally, 124,114 unigenes were identified by redundancy filtering, including 57,461 (46.3%), 26,025 (20.97%), 24,913 (20.07%), 54,901 (44.23%), and 43,369 (34.94%) unigenes collected from the homolog function annotation (*E* < 10^–5^) in the non-redundant (NR), Gene Ontology (GO), Kyoto Encyclopedia of Genes and Genomes (KEGG), eggNOG, and Swis-Sprot databases, respectively (Supplementary Tables 2, 3). Principal component analysis (PCA) and correlation evaluation showed that gene expression levels were clustered together among three replicates and distinguishable at 0, 24, and 72 hpi (Supplementary Figures 1A,B), indicating the reliability of the experimental design.

**FIGURE 3:**
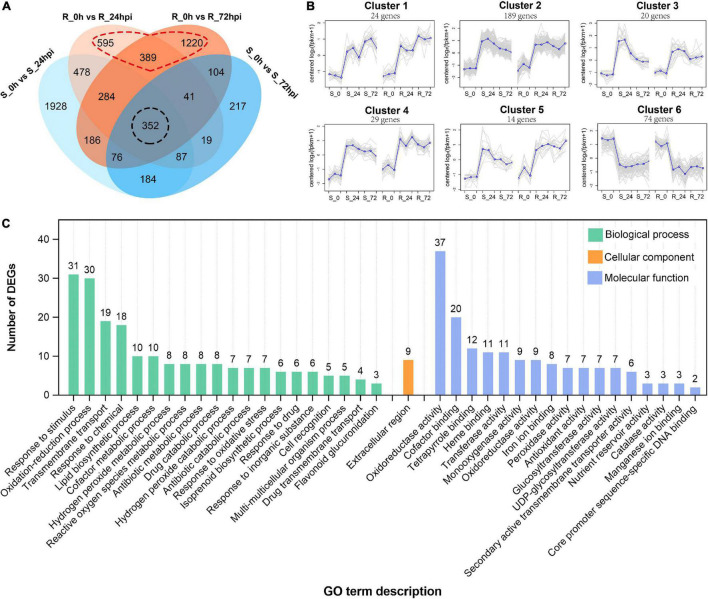
Differential expressed genes in susceptible (S) and resistant (R) *Cyclocarya paliuru*s cultivars after *Colletotrichum fructicola* infection. **(A)** Venn diagram of DEGs in R and S cultivars at 24 and 72 hpi, respectively; **(B)** cluster analysis of the (350/352) shared genes in both R and S cultivars. The blue line indicates the average expression level of all DEGs in different samples in a cluster, and the gray lines indicate the expression level of each DEG in different samples in a cluster. **(C)** GO functional classification of shared DEGs in both R and S cultivars.

Subsequently, 2,245 and 2,652 differential expressed genes (DEGs) (| log_2_ fold change| > l and FDR < 0.05) were identified from the resistance cultivar at 24 or 72 hpi versus the mock sample (0 hpi), respectively; and 3,575 and 1,080 DEGs were identified from the susceptible cultivar of the same comparisons (Supplementary Figures 1C,D). Among these, 352 DEGs are common expressions to both cultivars (Figure 3A), suggesting that both resistant and susceptible cultivars of *C. paliurus* have a similar defense network in response to *C. fructicola* infection. Gene expression clustering showed that (350/352) DEGs exhibited similar expression patterns between resistance and susceptible cultivars, most of which (276/350) were upregulated in response to *C. fructicola* infection (Figure 3B). GO analysis showed that the common DEGs were enriched in terms related to reduction–oxidation or biotic stress responses, which confirmed that both the resistant and susceptible cultivars should employ ROS burst against anthracnose (Figure 3C).

### Defense Network of Resistant *Cyclocarya paliurus* Responses to *Colletotrichum fructicola*

**FIGURE 4:**
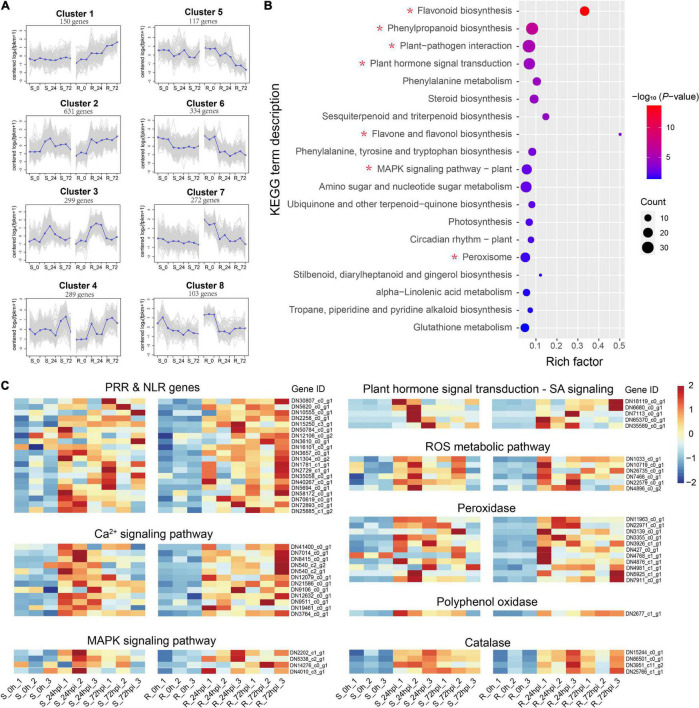
Differential expressed genes in resistant (R) *Cyclocarya paliurus* cultivar. **(A)** Cluster analysis of expression patterns. The blue line indicates the average expression level of all DEGs in different samples in a cluster, and the gray line indicates the expression level of each DEG in different samples in a cluster; **(B)** KEGG classifications of 2204 DEGs specific to R cultivar; **(C)** heatmap of 63 key DEGs upregulated in R cultivar.

Of the total DEGs, 2,204 genes were specifically differentially regulated in the resistant *C. paliurus* cultivar response to *C. fructicola* (Figure 3A), thus we employed these DEGs to describe the defense network of resistant *C. paliurus* against *C. fructicola*. Analysis of gene expression pattern showed that four gene clusters (a total of 1,369 genes) were expressed stronger in resistant cultivar than in susceptible cultivar response to *C. fructicola* (Figure 4A). GO analysis showed that DEGs were mainly enriched in functional oxidation–reduction processes, which are likely associated with the stress response; and also significantly enriched in many secondary metabolic processes, including polysaccharide metabolism, terpenoid biosynthesis, terpenoid metabolism, and flavonoid metabolism (Supplementary Figure 2). KEGG annotation showed that the DEGs mainly clustered into the functional pathways associated with disease resistance were highly enriched (*P* < 0.05), including plant-pathogen interaction, plant hormone signal transduction, phenylalanine metabolism, MAPK signaling pathway, and peroxisome (Figure 4B). Especially, the DEGs were extremely enriched in the flavonoid biosynthesis pathway (*P* = 2.12 × 10^–14^) (Figure 4B), which suggested that this pathway should play a critical role in *C. paliurus* resistance to *C. fructicola*. Heatmap visualization of gene expression patterns showed that the DEGs of these pathways were significantly upregulated in the resistant cultivar compared to no change or low expression levels in the susceptible cultivar during pathogen infection, including pattern-recognition receptors (PRRs) or nucleotide-binding leucine-rich repeat receptor (NLR) (20 DEGs), Ca^2+^ signaling (12 DEGs), ROS metabolic (6 DEGs), MAPK signaling (4 DEGs), and SA signaling (5 DEGs) (Figure 4C and Supplementary Table 4). In addition, 16 DEGs involved in defense enzyme, including 11 POD, 1 PPO, and 4 CAT, were also expressed higher in the resistant cultivar than in the susceptible cultivar (Figure 4C and Supplementary Table 4). Together, these results suggest that the resistance cultivar of *C. paliurus* employs a stronger defense response network against the *C. fructicola* than the susceptible cultivar.

### Metabolite Profiling Displays the Anthracnose Resistance Function of Flavonoids

With the strong activation of the metabolic network in *C. paliurus* resistance cultivar against *C. fructicola*, we further employed the widely targeted metabolome profiling to the resistance function role of metabolites in *C. paliurus*. The leaf samples were collected from resistant or susceptible *C. paliurus* cultivar as the treatment (24 and 72 hpi) for transcriptome analysis, with three independent biological replicates. Correlation and three-dimensional PCA plot analyses showed that the replicate of resistant or susceptible cultivar samples was well-clustered and distinguished from each other (Supplementary Figures 3A,B), indicating that substance accumulations of *C. paliurus* uniformly responded to *C. fructicola* infection at the metabolomic level for each replicates. In total, 365 differentially accumulated metabolites (DAMs) were detected from all the samples, of which 145 were metabolomic with known annotation that were also detected in these samples (Supplementary Table 5).

**FIGURE 5:**
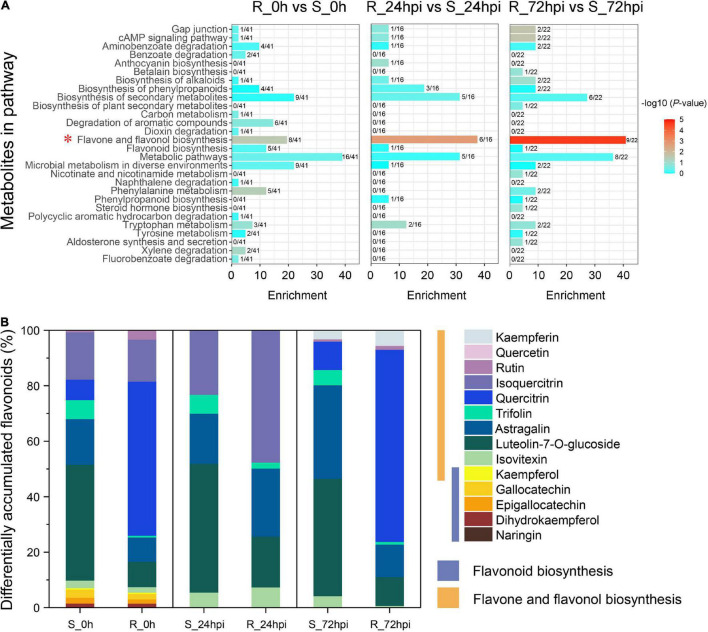
Metabolites differentially detected between resistant and susceptible cultivars at 0 h (before inoculation), 24, and 72 hpi. **(A)** KEGG pathway analysis. The *x*-axis represents the annotated metabolites in each pathway. The fractional number denotes the portion of the differentially accumulated metabolites in each pathway. **(B)** Compound analysis in a flavonoid-related pathway.

Investigation of the DAMs showed that the metabolites were significantly influenced by *C. fructicola* infection in the resistant cultivar compared to the susceptible cultivar at 24 and 72 hpi [variable importance of the projection (VIP) ≥1; Figure 5A and Supplementary Table 5]. Orthogonal projections to latent structures discriminant analysis (OPLS-DA) showed that 41, 16, and 22 DAMs were enriched in several known pathways between the resistant and susceptible cultivars in three comparison groups of mock inoculation (0 hpi), 24, and 72 hpi, respectively; which mainly involves in the aminobenzoate degradation, phenylpropanoids biosynthesis, flavone and flavonol biosynthesis, flavonoid biosynthesis, phenylalanine metabolism, and phenylpropanoid biosynthesis (Figure 5A). In particular, the flavone and flavonol biosynthesis pathway of *C. paliurus* significantly recruited many DAMs during infection with *C. fructicola* (mock group, *P* = 0.0282; 24 hpi group, *P* = 0.0019; and 72 hpi group, *P* = 2.37 × 10^–5^), in which nearly half DAMs converge into this pathway (Figure 5A). Further, analysis of the metabolites composition showed that the DAMs were mainly associated with the substances of flavonoid biosynthesis (naringin, dihydrokaempferol, epigallocatechin, etc.) in mock-inoculation, but highly enriched the substances of flavone and flavonol biosynthesis pathway (rutin, quercetin, kaempferin, etc.) when infected with *C. fructicola* (Figure 5B). Together, the results metabolite profiling further strongly suggested that the flavonoid-related pathways play critical role in the resistance anthracnose disease of *C. paliurus*.

### *Cyclocarya paliurus* Reprograms of Flavonoid Biosynthesis Pathway Against *Colletotrichum fructicola*

**FIGURE 6:**
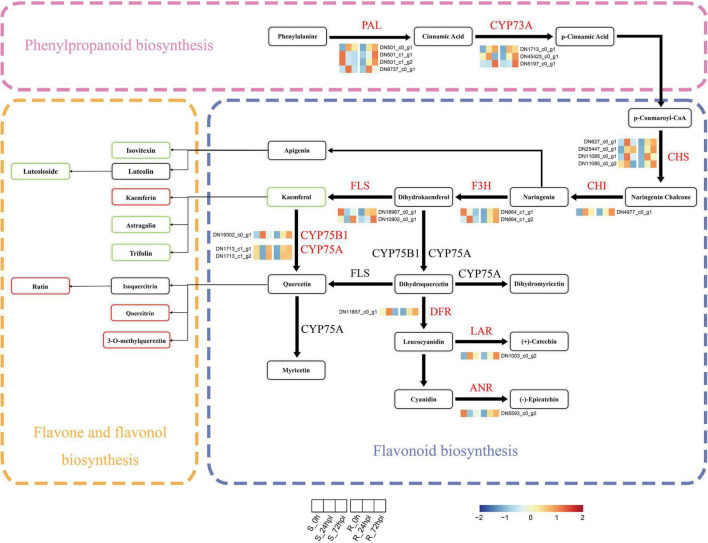
Expression profiles of genes and metabolites involved in the phenylpropanoids and flavonoid biosynthesis in resistant and susceptible *Cyclocarya paliurus* cultivars following *Colletotrichum fructicola* infection. Heatmap of relative expression levels of known genes encoding enzymes of the phenylpropanoid or flavonoid biosynthesis based on FPKM expression values. The metabolite rectangle patterns in red or green indicate significantly upregulated or downregulated metabolites in the R cultivar compared to the S cultivar at 72 hpi, respectively. PAL, phenylalanine ammonia-lyase; CYP73A, trans-cinnamate 4-monooxygenase; CHS, chalcone synthase; CHI, chalcone isomerase; F3H, flavanone 3-hydroxylase; FLS, flavonol synthase; CYP75B1, flavonoid 3′-monooxygenase; CYP75A, flavonoid 3′,5′-hydroxylase; DFR, dihydroflavonol 4-reductase; LAR, leucocyanidin reductase; ANR, anthocyanin reductase.

Next, we performed the integration of flavonoid biosynthesis-related pathway between transcriptome and metabolome data, including phenylpropanoid biosynthesis, flavonoid biosynthesis, and flavone-flavonol biosynthesis, to gain insight into the relationship between DAMs and DEGs in *C. paliurus* response to *C. fructicola* infection. Of the transcriptome data, 22 DEGs associated with flavonoid biosynthesis-related pathway, including PAL, trans-cinnamate 4-monooxygenase (CYP73A), chalcone synthase (CHS), 1 chalcone isomerase (CHI), naringenin 3-dioxygenase (F3H), flavonol synthase (FLS), flavonoids 3′,5′-hydroxylase (CYP75A), flavonoid 3′-monooxygenase (CYP75B1), anthocyanidin reductase (ANR), dihydroflavonol 4-reductase/flavanone 4-reductase (DFR), and leucoanthocyanidin reductase (LAR), were significantly upregulated in the resistant cultivar but downregulated or relatively low upregulated in the susceptible cultivar compared to control at both 24 and 72 hpi (Figure 6 and Supplementary Table 4). Random verification by RT-qPCR confirmed the expression pattern of four genes (PAL, CHI, FLS, and LAR) between the resistance and susceptible cultivar (Supplementary Figure 4). Correspondingly in metabolome data, several metabolites from flavones and flavonol biosynthesis metabolites were differentially accumulated at 72 hpi, including the increasing proportion of kaempferin, rutin, quercitrin, and 3-O-methylquercetin in resistance cultivar, and decreasing proportion of isovitexin, luteoloside, astragalin, and trifolin (Figures 5B, 6). Therefore, the integrated analysis of transcriptomics and metabolomics further suggested that the flavonoid pathway plays a critical role in *C. paliurus* resistance to *C. fructicola*.

### *Cyclocarya paliurus* Flavonoids Extraction Presents an Antifungal Activity to *Colletotrichum fructicola*

**FIGURE 7:**
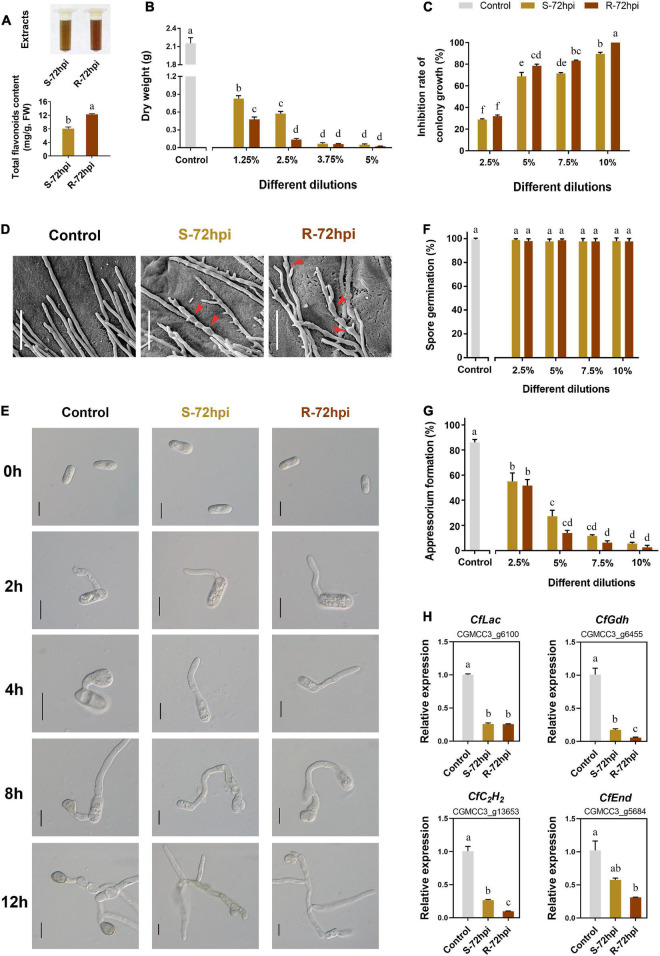
Antifungal activity of flavonoids (CPFs) extract of *Cyclocarya paliurus* from resistant and susceptible cultivars. **(A)** Extracts of CPFs (up) and total flavonoid content determination (below). Effect of total CPFs on *Colletotrichum fructicola* **(B)** fungal biomass; **(C)** colony growth; **(D)** mycelium microstructure (SEM); bars, 30 μm; **(E)** conidia germination and appressorium development progress; bars, 10 μm; **(F)** present of conidia germination; **(G)** appressorium formation; and **(H)** appressorium formation-related gene expression. Error bar on each column represents the standard error (*n* = 3). Columns with the same letter do not differ significantly according to Tukey’s test (*P* < 0.05).

To confirm the resistance function of flavonoids, the antifungal activity of CPFs extraction was evaluated at 72 hpi with *C. fructicola*. Quantitative analysis showed that the content of CPFs from resistant cultivars is significantly higher than the susceptible cultivar (Figure 7A). Detection of antifungal activity showed that flavonoids can strongly inhibit the mycelial growth and fungal biomass accumulation of *C. fructicola* (Supplementary Figure 5). Colony growth on the potato dextrose agar (PDA) medium plates showed that CPFs significantly inhibited *C. fructicola* biomass at low concentrations (<5.0%) (Figure 7B), and the *C. fructicola* completely lost mycelium growth under the 10% dilution of CPFs from the resistant cultivar at 5 days after incubation (*P* < 0.01) (Figure 7C and Supplementary Figure 5). Scanning electron microscopy showed that CPFs caused swelling of *C. fructicola* hyphal internodes, which probably affected its intracellular osmotic pressure (Figure 7D). Conidia germination and appressorium formation assays showed that CPFs did not inhibit conidia germination (Figures 7E,F), but the appressorium formation was significantly inhibited and the appressoria showed defective formation and melanization after conidia germination (Figures 7E,G). Moreover, RT-qPCR analysis showed that the transcript level of four appressorium formation-related genes, laccase (*CfLac*), endocytosis (*CfEnd*), glutamate dehydrogenase (*CfGdh*), and C_2_H_2_ transcription factor (*CfC_2_H_2_*), were significantly suppressed under the treatment with CPFs, which further evidenced the role of CPFs to inhibit appressorium formation in *C. fructicola* (Figure 7H). Together, these results further suggested that *C. paliurus* employs the antifungal activity of flavonoids against the infection of *C. fructicola*.

## Discussion

Outbreaks of *C. paliurus* anthracnose have occurred frequently in recent years, resulting in great yield and quality losses (Zheng et al., 2021). In this study, we screened two *C. paliurus* cultivars exhibiting a significantly varying degree of resistance to anthracnose (Figure 1A and Supplementary Table 1). By combining multi-omics and functional profiling analyses, we discovered the key components underlying anthracnose resistance mechanisms in *C. paliurus* and gained novel insights into CPF and their antifungal activity.

The plant’s innate immune system encodes NLRs proteins, which recognize effector proteins secreted by adapted pathogens (Cui et al., 2015; Zhang et al., 2017). Extensive previous studies have demonstrated that effector-triggered immunity (ETI) triggered by NLRs can stimulate HR (Wang et al., 2015; Hatsugai et al., 2017; Balint-Kurti, 2019; Pitsili et al., 2020; Dalio et al., 2021). However, recent studies have shown that plasma membrane PRRs coactivation could result in HR or enhance NLR-mediated HR cell death (Ngou et al., 2021; Yuan et al., 2021), given the fact that overexpressing PRRs coreceptor *AtBAK1* in *Arabidopsis thaliana* leads to an HR phenotype and higher resistance to hemibiotrophic pathogens (Domnguez-Ferreras et al., 2015). In our study, 20 DEGs annotated as NLRs or PRRs were significantly induced by *C. fructicola* in the resistant *C. paliurus* cultivar (Figure 4C and Supplementary Table 4). However, although the expression of these genes was upregulated in the susceptible cultivar at 24 hpi, their expression levels dropped sharply at 72 hpi (Figure 4C and Supplementary Table 4). Interestingly, DEGs related to Ca^2+^ and MAPK signaling pathways also presented a similar expression pattern (Figure 4C and Supplementary Table 4), which may indicate that the continuous expression of these resistant genes could play a role in HR. Accordingly, HR could result from these defense responses of the resistant *C. paliurus* to *C. fructicola*, as occurred in *Camellia sinensis* (Wang et al., 2018).

Transcriptome and metabolome are powerful tools for understanding cellular responses under biotic stress and revealing the functions of genes and metabolites. Our integration of transcriptomic and metabolic data revealed differential regulation of the flavonoid pathway in both resistant and susceptible *C. paliurus* cultivars in response to *C. fructicola* ingression (Figures 5A,B, 6). Indeed, DAMs involved in flavonoid biosynthesis are particularly interesting due to their association with differentially expressed flavonoid biosynthesis genes identified in the resistant cultivar (Figure 4B). Specifically, these results indicate that reprogramming and upregulation of the flavonoid pathway affect metabolic flux toward downstream flavone and flavonol biosynthesis (Figures 5B, 6). Flavonoid biosynthesis and metabolism pathways are critical for plant defenses through their antifungal activity. Similarly, knockdown of the *CHS* gene in *Casuarina glauca* reduces flavonoid levels, resulting in severely impaired nodulation (Abdel-Lateif et al., 2013). While exogenous flavonoid application enhances wheat resistance to *Fusarium graminearum* (Su et al., 2021).

Flavonoids also function as ROS scavengers or antioxidant molecules in preventing oxidative damage imposed by *Colletotrichum* or abiotic environmental stresses (Nakabayashi et al., 2014). Thus, transgenic expression of a flavonoid biosynthesis gene in sorghum (*Sorghum bicolor*) alleviated the oxidative damage caused by anthracnose (Wang L. et al., 2020). In the present study, ROS burst was visualized by histochemical staining and validated in the transcriptome, physiological, and qPCR assays (Figures 2A,B, 4C and Supplementary Figure 4). We suggest that flavonoids may play an important role in scavenging intracellular ROS that contributes to anthracnose resistance in *C. paliurus*. Together, our results suggest that the flavonoid pathway may play a role in the resistance of *C. paliurus* against *C. fructicola*.

The medicinal properties of flavonoids have been widely studied, including treatments for Alzheimer’s disease, Parkinson’s disease, and cancer (Panche et al., 2016; Braidy et al., 2017). Phytopathogen suppression by phytoalexin as flavonoids has recently become a hot topic, providing new avenues of research toward the prevention and management of anthracnose. Sakuranetin is a major phytoalexin flavonoid identified from rice (*Oryza sativa*) that has been shown to inhibit plant pathogens such as *Pyricularia oryzae*, *Gibberella fujikuroi*, and *Bipolaris oryzae* (Murata et al., 2020). Tangeretin substantially delays the formation of *Magnaporthe oryzae* appressoria, preventing rice blast (Liang et al., 2021). Similarly, sorghum produces flavones as a phytoalexin defense against anthracnose (Ibraheem et al., 2015). In addition, 3-deoxyanthocyanidin have antifungal activity against *Colletotrichum falcatum* (Nandakumar et al., 2020). In this study, CPFs strongly inhibited mycelial growth and fungal biomass in *C. fructicola* (Figures 7B,C and Supplementary Figure 5) and exhibited a significant antifungal effect on *C. fructicola* appressorium formation and melanization (Figures 7E,G,H).

Appressorium development is essential to penetrate the host. To explore the underlying mode of action of CPF’s effect on appressorium formation, we tested four genes reported to participate in the regulation of appressorium formation in *Colletotrichum* species (Miyara et al., 2010; Lin et al., 2012; Dubey et al., 2016; Wang et al., 2021). The expression of these appressorium formation-related genes was significantly suppressed by CPF treatment (Figure 7H). Corresponding to the phenotype of swelling of *C. fructicola* hyphal internodes in response to CPFs treatment, we speculate that CPFs may affect the ion transport in *C. fructicola*, which requires further research. Together, our results reveal that CPFs exhibit strong antifungal activity. However, the identification of specific metabolites responsible for antifungal activity requires further study.

## Materials and Methods

### Plant Materials, Pathogen Inoculation, and Sampling

Six natural *C. paliurus* cultivars were used from the origin of Anji, Zhejiang Province; Jinzhongshan, Guangxi Province; Jinggangshan, Jiangxi Province; Liping, Guizhou Province; Muchuan, Sichuan Province; and Wufeng, Hubei Province, respectively (Supplementary Table 6). Two-year-old seedlings of these cultivars were kindly provided by the *C. paliurus* Germplasm Resource Bank (CPGRB) in Baima, Jiangsu Province, China. The seedlings were surface-disinfected and maintained in an environmentally controlled greenhouse at 25°C and 90 ± 5% relative humidity under natural sunlight before pathogen inoculation.

The pathogenic strain used for inoculation was *C. fructicola* strain BM5, the predominant pathogen of *C. paliurus* anthracnose (Zheng et al., 2021). Before inoculation, *C. fructicola* was maintained on a potato dextrose agar (PDA) plate as previously described (Zheng et al., 2021). Conidial suspensions of *C. fructicola* were prepared as previously described (Fang et al., 2018) and diluted to 10^6^ conidia/mL with double-distilled water (ddH_2_O). To evaluate the resistance of the six *C*. *paliurus* cultivars to anthracnose, we performed DLI and IPI according to the methods of previous studies (Wang Z. H. et al., 2017; Zheng et al., 2021). In brief, for DLI, a PDA plug (5 mm in length) containing actively growing mycelia of *C. fructicola* was placed on a non-wounded detached leaf of *C. paliurus*; for IPI, a 100-mL conidia suspension (10^6^ conidia/mL) was sprayed onto an intact 2-year-old *C. paliurus* seedling. Detached leaves and seedlings treated with the non-colonized PDA plug or ddH_2_O were used as controls. The experiment was performed in triplicate for each treatment and control, involving five leaves or seedlings per replicate.

All inoculated seedlings or detached leaves were maintained in a greenhouse or transparent containers (25°C; 95–100% in humidity) as previously described (Zheng et al., 2021), with phenotypic changes in each cultivar recorded by photography. Lesion development, infection incidence (%), and disease severity (index of 0 to 5, 0 – no disease, 1 – mild disease, 5 – severe disease) were monitored and evaluated up to 14 days post-inoculation (dpi) (Jiang et al., 2019). Leaves (1 g in weight) were harvested from seedlings of Wufeng and Jinggangshan cultivars by IPI at 0 (before inoculation), 24, and 72 h post-inoculation (hpi) with *C. fructicola*, respectively. Three biological replicate sets of leaf samples were immediately frozen in liquid nitrogen and stored at −80°C before RNA extraction, physiological measurements, and metabolomic analyses. Each biological replicate consisted of leaves from two different seedlings.

### Physiological Measurements and Histochemical Staining

Frozen leaf samples, as aforementioned, were used to determine the activity of defense enzymes, phytohormone, and H_2_O_2_ content. PAL, CAT, PPO, and POD activity levels were determined using defense enzyme assay kits according to the manufacturer’s instructions (Solarbio, cat nos. BC0210, BC0200, BC0090, and BC0195, Beijing, China). H_2_O_2_ levels were measured by monitoring the titanium–peroxide complex (Tiryaki et al., 2019). SA, JA, and ABA contents were determined using ultra-performance liquid chromatography-electrospray ionization–tandem mass spectrometry (UPLC–ESI–MS/MS) in a facility at Lu Ming Biotechnology (Shanghai, China) as described by Liu et al. (2019). For each assay, three biological replicates were conducted.

Leaves were harvested from *C. paliurus* seedlings at 0 (before inoculation) and 24 hpi after IPI as described earlier, and were used for superoxide (O_2_⋅**^–^**) and H_2_O_2_ staining. Nitroblue tetrazolium chloride (NBT; Sangon, Shanghai, China) and 3,3-dimethoxybenzidine (DAB; Sangon, Shanghai, China) were used to visualize O_2_⋅**^–^** and H_2_O_2_ accumulation, respectively, following the histochemical protocols of Wang H. et al. (2017), with minor modification. In brief, leaf samples were soaked in 0.25 mM NBT or 1 mg/mL DAB (pH 3.8) solution, and then vacuum infiltrated for 30 min. After 6 h, the samples were washed with ddH_2_O and decolorized in heated 99% ethanol until the leaves no longer contained chlorophyll, and O_2_⋅**^–^** and H_2_O_2_ levels were visualized in leaves as the intensity of blue insoluble formazan or brown polymerization product, respectively.

### RNA Extraction, cDNA Library Construction, and Sequencing

Total RNA was extracted from *C. paliurus* leaves using the E.Z.N.A. Plant RNA Kit (Omega Bio-Tek, United States). RNA quality was assessed *via* gel electrophoresis and the concentration was evaluated using a Qubit fluorometer (Thermo Fisher Scientific, Wilmington, NC, United States) and Nanodrop 2000 spectrophotometer (Thermo Fisher Scientific, Wilmington, NC, United States). A total of 18 high-quality RNA samples (three collection times in resistant or susceptible cultivars, with three biological replicates) were prepared for cDNA library construction. Sequencing libraries were generated using the TruSeq RNA Sample Preparation Kit (Illumina, San Diego, CA, United States). First, mRNA was purified from total RNA using poly T oligo attached magnetic beads. Fragmentation was carried out using divalent cations under elevated temperature in an Illumina proprietary fragmentation buffer. First-strand cDNA was synthesized using random oligonucleotides and SuperScript II. Second strand cDNA synthesis was subsequently performed using DNA Polymerase I and RNase H. Transcriptome sequencing was performed to obtain paired-end reads (150 bp in length) on the Illumina HiSeq 2500 platform by Personal Biotechnology (Shanghai, China). Raw sequencing data were uploaded to the National Center for Biotechnology Information (NCBI) Sequence Read Archive database under project no. PRJNA783768.

### Filtration and Transcriptomic Analysis

After adaptor sequences and low-quality reads were removed (Liu et al., 2019), the remaining clean reads were mapped to the genome of *C. fructicola* CGMCC 3.17371 (GenBank accession no. SSNE00000000) for the exclusion of reads that contained *C. fructicola* sequences. The pathogen-sequence-free reads (*C. paliurus*) were *de novo* assembled using the Trinity v2.5.1 software. Functional annotation of each unigene was performed based on NCBI NR, eggNOG, Swiss-Prot, KEGG, and GO databases at a threshold of *E* < 10^–5^. The expression level of each unigene was calculated based on the fragments per kilobase of exon per million fragments mapped (FPKM) using the RSEM software. Raw counts were obtained for each unigene and differentially expressed genes (DEGs) between groups were analyzed using the *DESeq* R package with a false discovery rate (FDR) < 0.05 and | log_2_ fold change| > 1. KEGG pathway and GO enrichment analyses were performed to screen DEGs with significantly enriched KEGG (*P* < 0.05) or GO (FDR < 0.05) terms according to the KOBAS and BLAST2GO software, respectively (Lu et al., 2020). A heatmap was created to visualize the expression patterns of DEGs between the treatment and control groups.

### Quantitative Reverse-Transcription Polymerase Chain Reaction Validation

To verify the transcriptome data and gene expression patterns, we investigated the relative expression of eight DEGs. From each RNA sample, cDNA was synthesized using Hifair III First Strand cDNA Synthesis SuperMix with gDNA Digester Plus (Yeasen Biotech, Shanghai, China), following the manufacturer’s instructions, and used as a template (Lu et al., 2020). Primers for qPCR were designed using the Primer Quest Tool software^1^ (Supplementary Table 7) according to the transcriptome data. qPCR was performed on an Applied Biosystems 7500 Real-Time PCR System (Thermo Fisher Scientific, Wilmington, NC, United States) with UNICON qPCR SYBR Green Master Mix (Yeasen Biotech, Shanghai, China), and the reaction procedure was performed following the manufacturer’s instructions. The comparative CT method (2^–ΔΔ*CT*^ method) was used to quantify gene expression using the *Cp18sRNA* gene as an endogenous control (Zhang et al., 2021). Three technical replicates and three biological replicates were conducted for each sample.

### Widely Targeted Metabolome Analysis

For further metabolomics analysis of secondary metabolites, 2-year-old *C. paliurus* seedlings of resistant (Wufeng) and susceptible (Jinggangshan) cultivars were inoculated following the IPI method as described earlier. The sample preparation, extract analysis, metabolite separation, and detection were conducted by MetWare Biological Science and Technology (Wuhan, China) following their standard procedures, which were previously described by Li et al. (2021) and Mei et al. (2020). In brief, 100 mg crushed, freeze-dried sample was extracted overnight at 4°C with 0.6 mL 70% (v/v) aqueous methanol. After centrifuging at 10,000 rpm for 10 min, the extracts were absorbed and filtered, and then analyzed on a UPLC–ESI–MS/MS system (UPLC, Shim-pack UFLC SHIMADZU CBM30A system; MS, Applied Biosystems 4500 QTRAP). Metabolite quantification was conducted utilizing the multiple reaction monitoring (MRM) method (Chen et al., 2013). Following data evaluation (quality control and PCA analysis), OPLS-DA, a supervised multivariate method, was used to maximize metabolome differences between sample pairs. Differentially accumulated metabolites (DAMs) were set at fold change (FC) > 2 or FC < 0.5 and OPLS-DA VIP ≥ 1. The identified DAMs were further annotated using the KEGG compound database to reveal the function and content variation of these metabolites.

### Flavonoid Extraction and Antifungal Activity Validation *in vitro*

*Cyclocarya paliurus* leaves of resistant and susceptible cultivars were inoculated using the IPI method and sampled at 72 hpi as described earlier; while uninoculated plants were used as mock controls. The extraction and quantification of total CPFs were performed using ultrasonic and colorimetric methods as previously described by Zhang et al. (2021) and Deng et al. (2015). In addition, a blank 70% (v/v) ethanol was subjected to the extraction procedure, serving as the solvent (control).

For the colony inhibition assay, mycelial disks (2 mm in diameter) aseptically collected from cultures of *C. fructicola* were transferred onto PDA plates (7 cm in diameter) amended with different dilutions of CPFs (10, 7.5, 5, and 2.5%) or 10% blank solvent (control), and inoculated at 25°C in the dark. Colony diameters were measured at 5 dpi, and mycelium morphology was observed under a Quanta 200 environmental scanning electron microscope (FEI, United States) following the method of Kong et al. (2020). The inhibition rate was calculated according to Ilboudo et al. (2016). For each treatment, three replicates were conducted, and the experiment was repeated twice.

For the fungal biomass assay, a conidia suspension of *C. fructicola* was prepared following the protocol of Kong et al. (2020), and then a 50-μL conidia suspension (10^8^ conidia/mL) was added to 200 mL liquid complete medium (CM) (Wang P. et al., 2020) amended with different dilutions of CPFs (5, 3.75, 2.5, and 1.25%) or 5% blank solvent (control), and inoculated on a shaker (25°C, 100 rpm). Mycelial biomass was estimated by weighing the mycelium after filtration on Whatman paper at 3 dpi. Three replicates were conducted for each treatment, and the experiment was repeated twice.

For conidia germination and appressorium formation assays, 10 μL drops of conidia suspension (10^5^ conidia/mL) containing different dilutions of CPFs (10, 7.5, 5, and 2.5%) or 10% blank solvent (control) were placed on a hydrophobic microscope slide (Fisher Scientific, United States) following the method of Wang P. et al. (2020). At 4 and 12 hpi, appressorium formation and conidia germination percentages were calculated under a light microscope (Carl Zeiss, Germany). Each treatment was repeated twice, and at least 100 conidia were analyzed per replicate.

### Effects of *Cyclocarya paliurus* Flavonoids on the Expression of Appressorium Formation-Related Genes in *Colletotrichum fructicola*

To further explore the effects of CPFs on appressorium formation in *C. fructicola*, we performed quantitative reverse-transcription polymerase chain reaction (qRT-PCR) of its related genes in *C. fructicola*. A conidia suspension of *C. fructicola* was prepared as described earlier. Next, 100 μL conidia suspensions (10^6^ conidia/mL) were coated onto PDA plates amended with 5% CPFs or 5% blank solvent (control) and incubated at 25°C in the dark for 60 hpi. Total RNA was extracted from mycelia grown on the PDA plates using TRIzol reagent (Yuanye Biotech, Shanghai, China) according to the manufacturer’s instructions. cDNA synthesis and qPCR reactions were conducted as described earlier. Four appressorium formation-related genes, *CfLac*, *CfEnd*, *CfGdh*, and *CfC_2_H_2_*, were selected for gene expression analysis (Supplementary Table 7). The *β-tubulin* gene was used as an endogenous reference (Shang et al., 2020). Relative quantification of changes in gene expression was conducted following the 2^–ΔΔCT^ method as previously described. Three independent biological replicates were involved in this experiment.

### Statistical Analyses

Data are presented as means ± standard error (SE). Differences between treatments were evaluated using independent-samples Student’s *t*-tests and one-way analysis of variance (ANOVA) in the GraphPad Prism v9.0 (GraphPad Software Inc., San Diego, CA, United States) and IBM SPSS Statistics 24.0 software, respectively (SPSS Inc., Chicago, IL, United States).

## Data Availability Statement

The datasets presented in this study can be found in online repositories. The names of the repository/repositories and accession number(s) can be found in the article/Supplementary Material.

## Author Contributions

F-MC and J-YC conceived and designed the research. X-RZ was responsible for the entire process of experimentation and writing the manuscript. M-JZ and Y-HQ helped perform the experiment and analyzed the results. RL edited the manuscript. All authors contributed to the article and approved the submitted version.

## Conflict of Interest

The authors declare that the research was conducted in the absence of any commercial or financial relationships that could be construed as a potential conflict of interest.

## Publisher’s Note

All claims expressed in this article are solely those of the authors and do not necessarily represent those of their affiliated organizations, or those of the publisher, the editors and the reviewers. Any product that may be evaluated in this article, or claim that may be made by its manufacturer, is not guaranteed or endorsed by the publisher.

## Footnotes

1
https://sg.idtdna.com/pages/tools
