# Supplementary material for: Cyclocarya paliurus Reprograms the Flavonoid Biosynthesis Pathway Against Colletotrichum fructicola
Source: Front Plant Sci. 2022 Jun 30;13:933484. doi: 10.3389/fpls.2022.933484 (PMC9280340; doi:10.3389/fpls.2022.933484)
Supplement: Supplementary file 1 [file Data_Sheet_1.docx]

## Supplementary Figures

### Figure S1


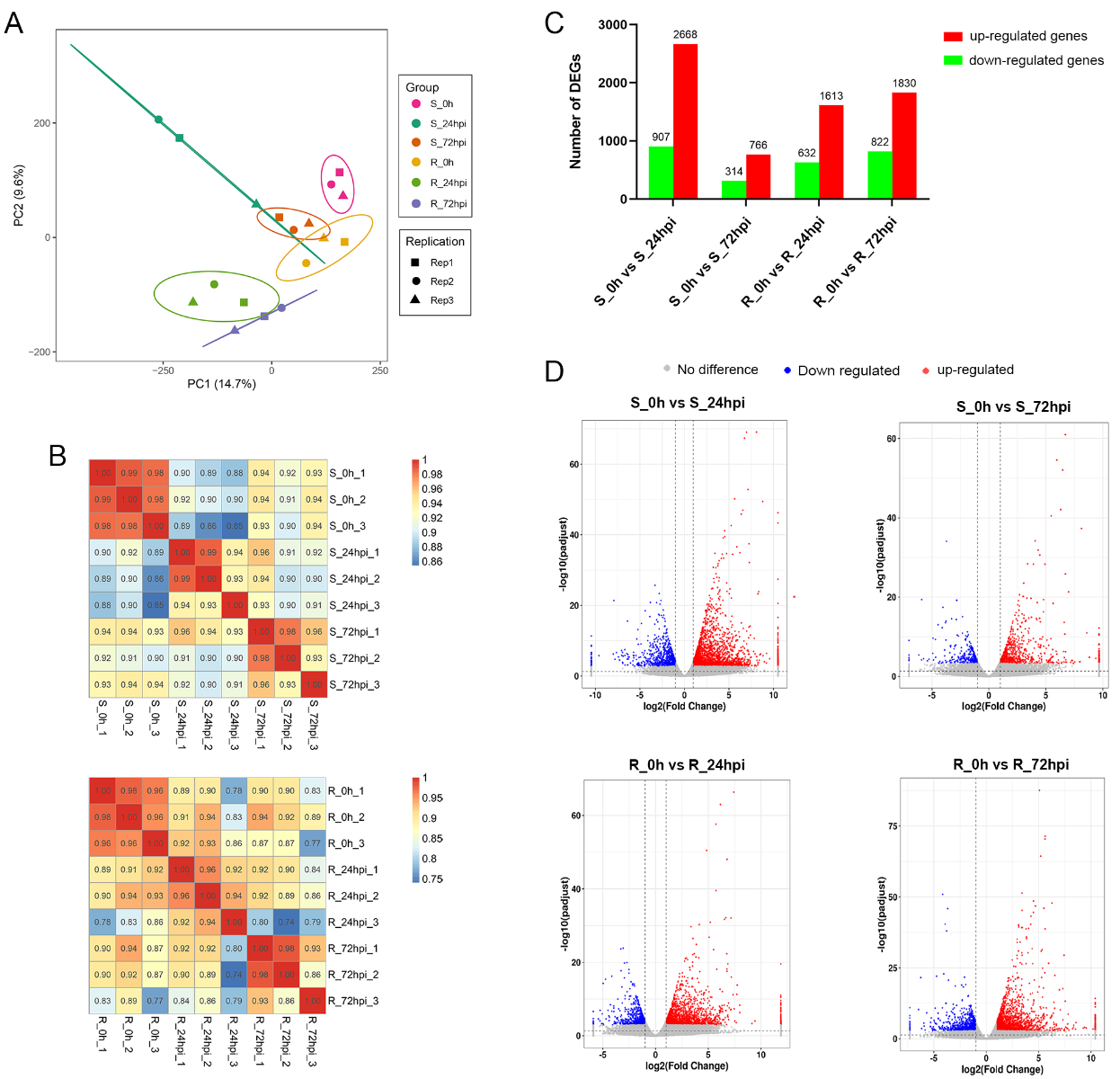


**Figure S1.** Overview of transcriptome analysis of resistant (R) and susceptible (S) *Cyclocarya paliurus* responsive to *Colletotrichum fructicola* infection at 0 h, 24 and 72 hours post infection (hpi). A. Principal component analysis and (B) Correlation evaluation analysis of gene expression levels. C. The histogram analysis of DEGs between S_0h vs S_24hpi, S_0h vs S_72hpi, R_0h vs R_24hpi, R_0h vs R_72hpi. D. Volcano plot analysis of DEGs between S_0h vs S_24hpi, S_0h vs S_72hpi, R_0h vs R_24hpi, R_0h vs R_72hpi.

### Figure S2


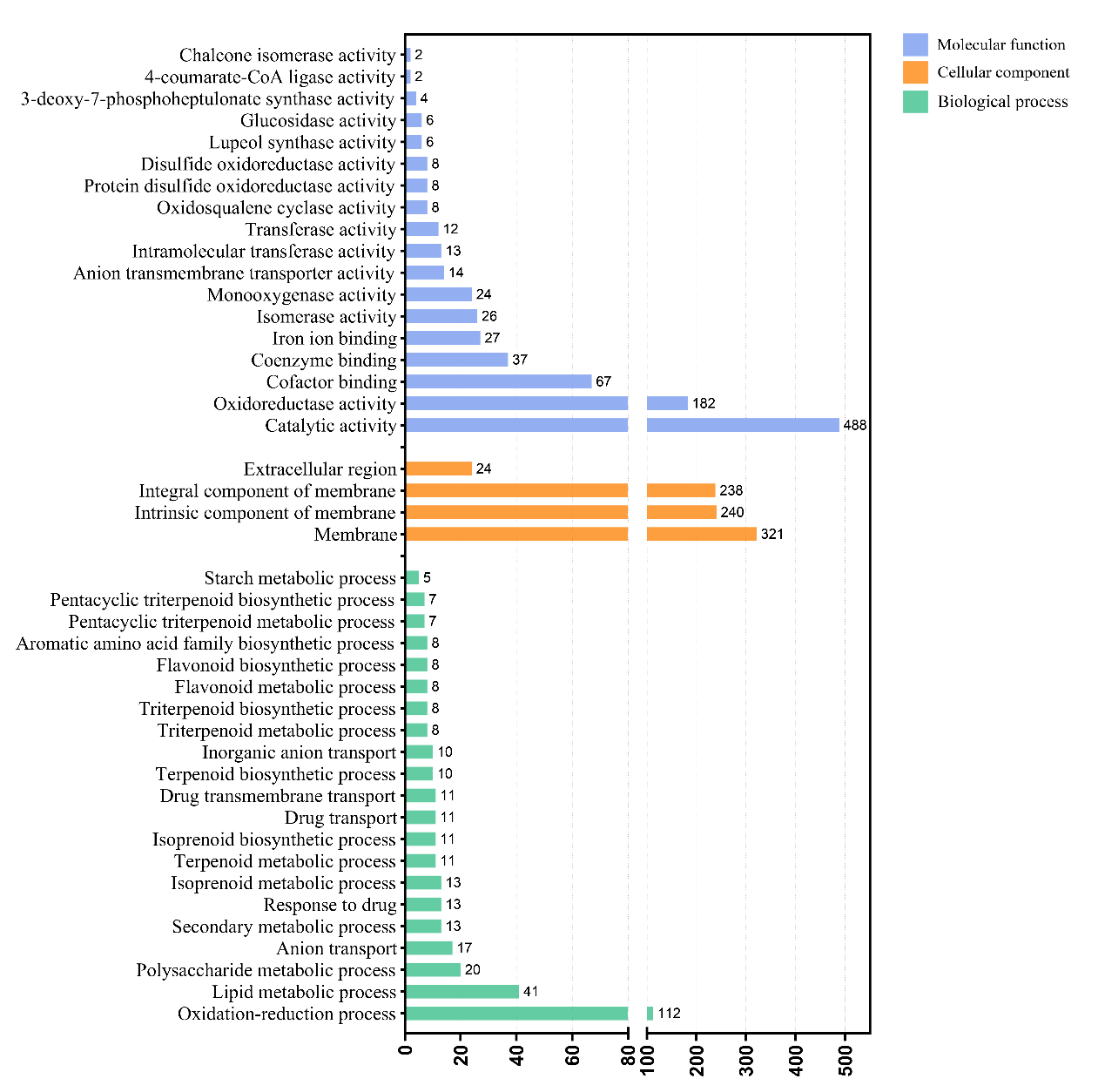


**Figure S2** GO functional classification of DEGs specific to resistant *Cyclocarya paliurus* cultivar.

### Figure S3


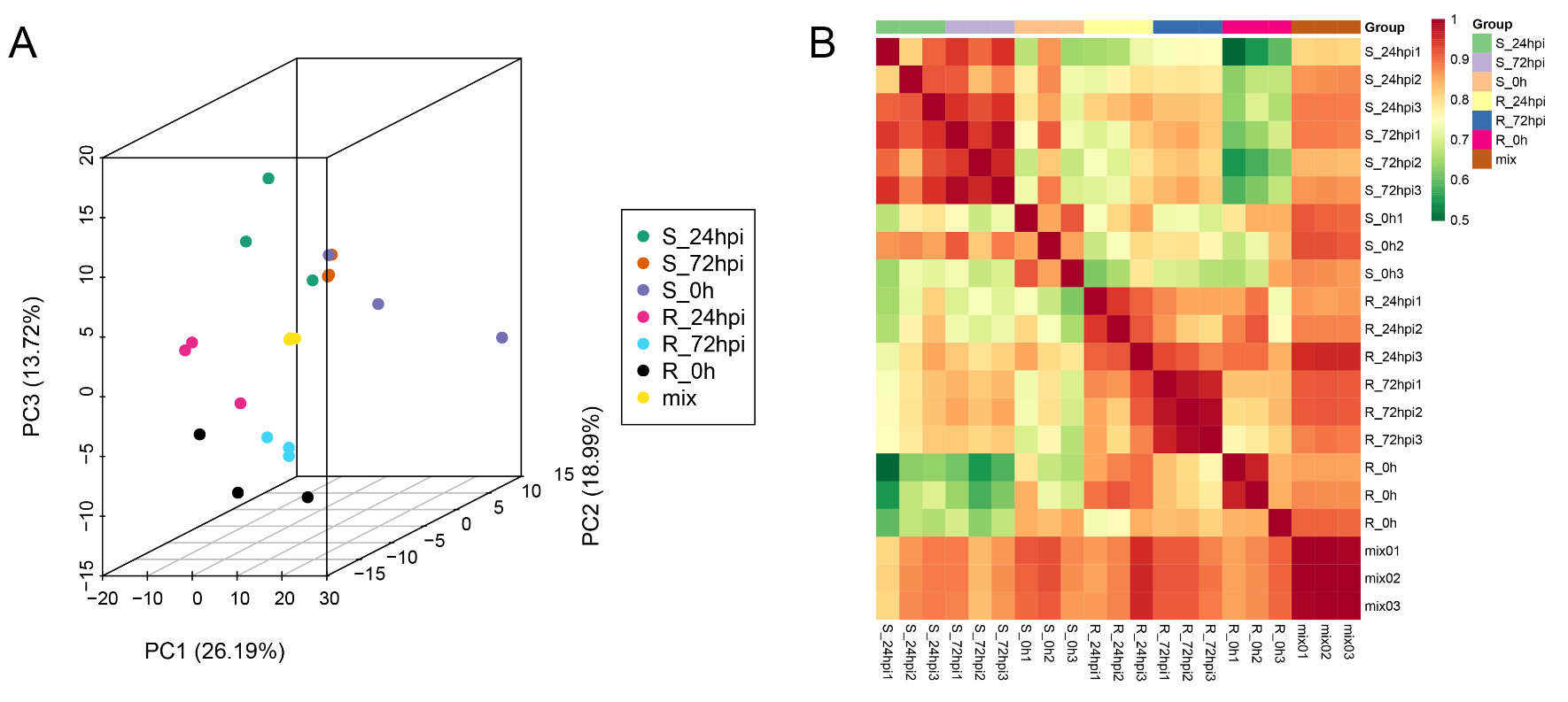


**Figure S3.** Overview of widely targeted metabolome analysis of resistant (R) and susceptible (S) *Cyclocarya paliurus* responsive to *Colletotrichum fructicola* infection at 0 h, 24 and 72 hours post infection (hpi). A. Three-dimensional principal component analysis of samples. B. Correlation evaluation analysis of metabolites among samples.

### Figure S4


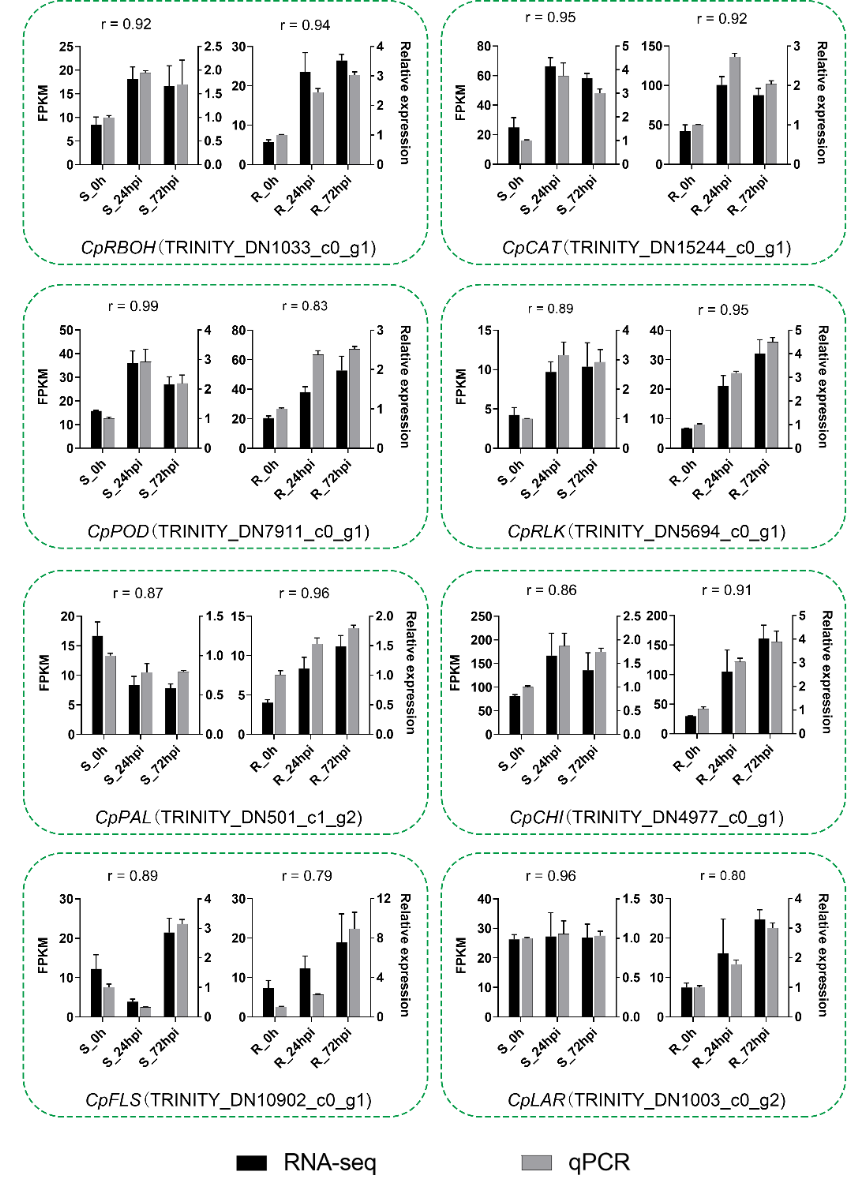


**Figure S4**. Expression profiling of eight DEGs identified in resistant (R) and susceptible (S) *Cyclocarya paliurus* responsive to *Colletotrichum fructicola* infection at 0 h, 24 and 72 hours post infection (hpi). The left *y*-axes show FPKM values determined by RNA-Seq, and the right *y*-axes show relative expression levels determined by qPCR. The *r* value representing the correlation between the qPCR and RNA-Seq results is listed in the top of each figure. The comparative CT method (2^–ΔΔCT^ method) was used to quantify gene expression using the *Cp18sRNA* gene as an endogenous control. Error bar on each column represents the standard error (three biological replicates, n=3).

### Figure S5


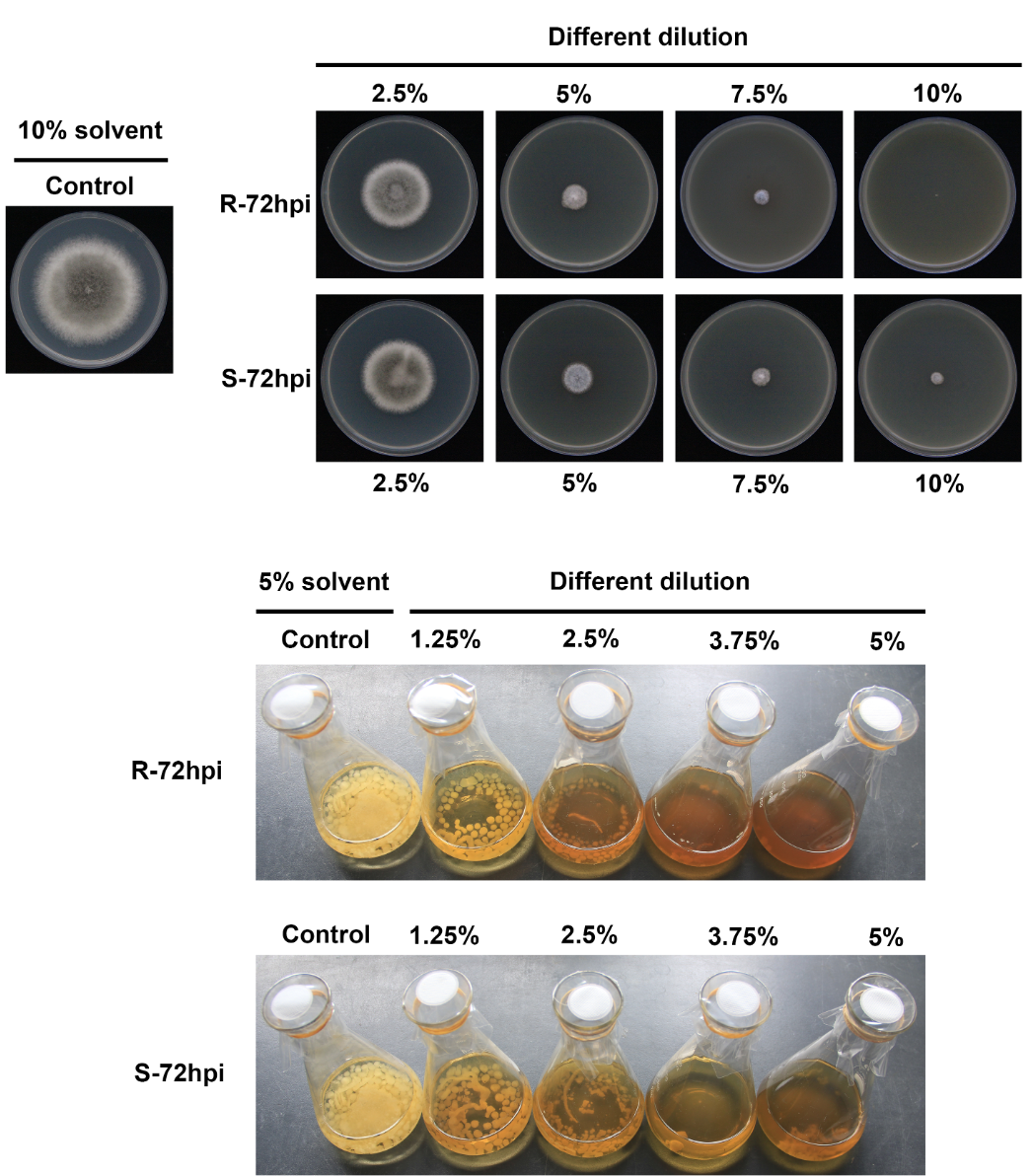


**Figure S5.** Effect of total flavonoids extracts of resistant (R) and susceptible (S) *Cyclocarya paliurus* on *Colletotrichum fructicola* colony growth (above) and fungal biomass (below), respectively. A blank 70% (v/v) ethanol was subjected to the extraction procedure, serving as the solvent.

| **Supplementary Tables**  **Table S1. Anthracnose severity and incidence of six *Cyclocarya paliurus* cultivars using intact plant inoculation.** | | |
| --- | --- | --- |
| Natural cultivars | Disease severity index | Disease incidence (%) |
| Anji | 0.65±0.10cd | 91.3±2.5bc |
| Jinggangshan | 0.83±0.07d | 97.3±2.5c |
| Jinzhongshan | 0.44±0.04bc | 71.3±5.7b |
| Lipin | 0.56±0.03bc | 78.7±1.9bc |
| Muchuan | 0.35±0.04ab | 74.0±5.9bc |
| Wufeng | 0.17±0.06a | 42.0±9.9a |
| Disease severity index and disease incidence were calculated from 150 leaves on seedlings at 14 days post-inoculation (dpi).  Data are mean ± standard error. Columns with the same letter do not differ significantly according to Tukey’s test (*P* < 0.01). | | |

| **Table S2. Quality of RNA-seq output data of resistant (R) and susceptible (S) *Cyclocarya paliurus* infected with** ***Colletotrichum fructicola* at 0 h, 24 and 72 hours post infection (hpi).** | | | | | | |
| --- | --- | --- | --- | --- | --- | --- |
| Sample | Raw Reads | Clean reads (Percentage/%) | Clean bases | N (%) | Q20 (%) | Q30 (%) |
| R_0h_1 | 47112668 | 43390190 (92.09) | 6.06G | 0.000506 | 97.27 | 93.03 |
| R_0h_2 | 45835626 | 42372208 (92.44) | 5.92G | 0.000513 | 96.95 | 92.38 |
| R_0h_3 | 43348404 | 40054026 (92.4) | 5.60G | 0.000512 | 97.31 | 93.09 |
| R_24hpi_1 | 45519964 | 42144324 (92.58) | 5.89G | 0.000483 | 97.27 | 92.99 |
| R_24hpi_2 | 40738962 | 37497638 (92.04) | 5.24G | 0.000504 | 97.27 | 93.02 |
| R_24hpi_3 | 47155538 | 43649698 (92.56) | 6.10G | 0.000510 | 97.08 | 92.63 |
| R_72hpi_1 | 43775610 | 40510166 (92.54) | 5.66G | 0.000509 | 97.15 | 92.8 |
| R_72hpi_2 | 44207712 | 40960616 (92.65) | 5.72G | 0.000497 | 97.35 | 93.18 |
| R_72hpi_3 | 41490012 | 38300486 (92.31) | 5.35G | 0.000493 | 97.32 | 93.12 |
| S_0h_1 | 47666738 | 43826052 (91.94) | 6.12G | 0.000504 | 97.22 | 92.92 |
| S_0h_2 | 42646690 | 39307848 (92.17) | 5.49G | 0.000500 | 97.22 | 92.9 |
| S_0h_3 | 44134320 | 40698212 (92.21) | 5.69G | 0.000495 | 97.48 | 93.46 |
| S_24hpi_1 | 43709178 | 40614682 (92.92) | 5.67G | 0.000500 | 97.28 | 93.02 |
| S_24hpi_2 | 43632134 | 40525360 (92.87) | 5.66G | 0.000493 | 97.43 | 93.34 |
| S_24hpi_3 | 46516392 | 42637638 (91.66) | 5.96G | 0.000501 | 97.41 | 93.3 |
| S_72hpi_1 | 46987678 | 43413012 (92.39) | 6.06G | 0.000519 | 97.31 | 93.11 |
| S_72hpi_2 | 41043436 | 37818424 (92.14) | 5.28G | 0.000509 | 97.12 | 92.78 |
| S_72hpi_3 | 44704486 | 41355022 (92.5) | 5.78G | 0.000504 | 97.23 | 92.96 |

| **Table S3. Number of reads of the *Cyclocarya paliurus* transcriptome**. | |
| --- | --- |
|  | Number |
| All transcripts | 336157 |
| All unigenes | 124114 |
| Unigenes annotated by NR | 57461 |
| Unigenes annotated by GO | 26025 |
| Unigenes annotated by KEGG | 24913 |
| Unigenes annotated by eggNOG | 54901 |
| Unigenes annotated by Swissprot | 43369 |
| Contig N50 (bp) | 1837 |
| Contig N90 (bp) | 508 |
| Total assembled bases (bp) | 406774316 |

| **Table S4. The DEGs involved in defense in *Cyclocarya paliurus* during *Colletotrichum fructicola* infection and used in a heatmap.** | | | | | |
| --- | --- | --- | --- | --- | --- |
| ID | Description | Fold change versus respective level at 0 h | | | |
|  |  | S provenance | | R provenance | |
|  |  | S_24hpi | S_72hpi | R_24hpi | R_72hpi |
| TRINITY_DN501_c0_g1 | Phenylalanine ammonia-lyase (PAL) | 1.6 | 1.4 | 17.7 | 20.8 |
| TRINITY_DN501_c1_g1 | Phenylalanine ammonia-lyase (PAL) | 0.6 | 0.6 | 1.4 | 2.3 |
| TRINITY_DN501_c1_g2 | Phenylalanine ammonia-lyase (PAL) | 0.5 | 0.5 | 2.1 | 2.7 |
| TRINITY_DN8737_c0_g1 | Phenylalanine ammonia-lyase (PAL) | 7.7 | 1.1 | 5.6 | 2.4 |
| TRINITY_DN1713_c0_g1 | Trans-cinnamate 4-monooxygenase (CYP73A) | 2.2 | 2.7 | 3.9 | 3.2 |
| TRINITY_DN45423_c0_g1 | Trans-cinnamate 4-monooxygenase (CYP73A) | 1.4 | 0.4 | 5.5 | 8.5 |
| TRINITY_DN5197_c0_g1 | Trans-cinnamate 4-monooxygenase (CYP73A) | 0.0 | 25.6 | 44.7 | 675.7 |
| TRINITY_DN627_c0_g1 | Chalcone synthase (CHS) | 1.4 | 1.1 | 17.9 | 21.6 |
| TRINITY_DN25447_c0_g1 | Chalcone synthase (CHS) | 0.9 | 1.1 | 9.0 | 14.6 |
| TRINITY_DN11085_c0_g1 | Chalcone synthase (CHS) | 26.9 | 7.1 | 16.1 | 7.9 |
| TRINITY_DN11085_c0_g2 | Chalcone synthase (CHS) | 1.1 | 0.5 | 11.4 | 19.2 |
| TRINITY_DN4977_c0_g1 | Chalcone isomerase (CHI) | 2.0 | 1.7 | 3.7 | 5.4 |
| TRINITY_DN1713_c1_g1 | Flavonoid 3',5'-hydroxylase (CYP75A) | 0.6 | 1.1 | 10.3 | 10.7 |
| TRINITY_DN1713_c1_g2 | Flavonoid 3',5'-hydroxylase (CYP75A) | 0.6 | 1.1 | 10.7 | 10.2 |
| TRINITY_DN19302_c0_g1 | Flavonoid 3'-monooxygenase (CYP75B1) | 1.7 | 1.2 | 5.2 | 6.7 |
| TRINITY_DN864_c1_g1 | Naringenin 3-dioxygenase (F3H) | 0.9 | 0.8 | 9.0 | 10.8 |
| TRINITY_DN864_c1_g2 | Naringenin 3-dioxygenase (F3H) | 1.3 | 1.0 | 8.0 | 10.5 |
| TRINITY_DN18967_c0_g1 | Flavonol synthase (FLS) | 0.7 | 0.8 | 6.9 | 6.3 |
| TRINITY_DN10902_c0_g1 | Flavonol synthase (FLS) | 0.3 | 1.8 | 1.6 | 4.0 |
| TRINITY_DN5593_c0_g2 | Anthocyanidin reductase (ANR) | 0.8 | 0.9 | 3.0 | 3.2 |
| TRINITY_DN11857_c0_g1 | Dihydroflavonol 4-reductase/flavanone 4-reductase (DFR) | 1.3 | 0.7 | 7.5 | 9.4 |
| TRINITY_DN1003_c0_g2 | Leucoanthocyanidin reductase (LAR) | 1.0 | 1.0 | 2.3 | 3.4 |
| TRINITY_DN30807_c0_g1 | Brassinosteroid insensitive 1-associated receptor kinase 1 | 3.6 | 8.2 | 4.9 | 6.5 |
| TRINITY_DN5620_c0_g1 | Brassinosteroid insensitive 1-associated receptor kinase 1 | 1.3 | 1.7 | 2.2 | 1.9 |
| TRINITY_DN10555_c0_g1 | Disease resistance protein RPM1 | 0.9 | 1.6 | 1.7 | 2.5 |
| TRINITY_DN2258_c0_g1 | Disease resistance protein RPM1 | 2.3 | 2.3 | 2.3 | 2.5 |
| TRINITY_DN15250_c3_g1 | Disease resistance protein RPM1 | 3.0 | 3.1 | 5.0 | 2.5 |
| TRINITY_DN50784_c0_g1 | Disease resistance protein RPS2 | 2.1 | 1.3 | 4.6 | 4.3 |
| TRINITY_DN12106_c0_g2 | Disease resistance protein RPS2 | 1.2 | 1.0 | 1.6 | 2.0 |
| TRINITY_DN3610_c0_g1 | Disease resistance protein RPS2 | 0.8 | 1.4 | 1.4 | 3.0 |
| TRINITY_DN16101_c0_g1 | Disease resistance protein RPS2 | 1.3 | 1.0 | 2.2 | 1.8 |
| TRINITY_DN3657_c0_g1 | LRR receptor kinase BAK1 | 3.6 | 2.0 | 3.6 | 3.3 |
| TRINITY_DN1304_c0_g2 | LRR receptor-like serine/threonine-protein kinase FLS1 | 6.2 | 2.5 | 4.9 | 5.7 |
| TRINITY_DN1781_c1_g1 | LRR receptor-like serine/threonine-protein kinase FLS1 | 1.5 | 2.4 | 2.1 | 2.6 |
| TRINITY_DN2729_c1_g1 | LRR receptor-like serine/threonine-protein kinase FLS1 | 1.9 | 2.1 | 2.2 | 2.0 |
| TRINITY_DN35058_c0_g1 | LRR receptor-like serine/threonine-protein kinase FLS1 | 1.5 | 2.7 | 1.7 | 2.0 |
| TRINITY_DN40267_c0_g1 | LRR receptor-like serine/threonine-protein kinase FLS1 | 1.3 | 2.0 | 1.9 | 2.7 |
| TRINITY_DN5694_c0_g1 | LRR receptor-like serine/threonine-protein kinase FLS1 | 2.1 | 2.5 | 3.1 | 4.5 |
| TRINITY_DN58172_c0_g1 | LRR receptor-like serine/threonine-protein kinase FLS1 | 3.0 | 1.9 | 1.2 | 2.0 |
| TRINITY_DN70619_c0_g1 | LRR-RLK | 3.3 | 2.5 | 2.5 | 1.8 |
| TRINITY_DN72893_c0_g1 | LRR-RLK | 4.8 | 3.2 | 15.6 | 14.4 |
| TRINITY_DN25885_c1_g2 | NBS-LRR type disease resistance protein | 2.2 | 1.2 | 1.4 | 2.0 |
| TRINITY_DN41400_c0_g1 | Calmodulin | 1.4 | 1.2 | 4.1 | 6.9 |
| TRINITY_DN7014_c0_g1 | Calmodulin | 6.1 | 1.4 | 2.8 | 3.0 |
| TRINITY_DN8415_c0_g1 | Calmodulin | 12.7 | 6.8 | 3.0 | 3.0 |
| TRINITY_DN540_c2_g2 | Cyclic nucleotide gated channel | 1.6 | 0.8 | 19.0 | 35.3 |
| TRINITY_DN540_c2_g1 | Cyclic nucleotide gated channel | 1.9 | 1.1 | 12.0 | 16.9 |
| TRINITY_DN12079_c0_g1 | Calcium-transporting ATPase 8 | 3.8 | 2.7 | 3.4 | 3.1 |
| TRINITY_DN21586_c0_g1 | Calcium-dependent protein kinase | 4.4 | 3.0 | 2.8 | 3.1 |
| TRINITY_DN9106_c0_g1 | Calcium-dependent protein kinase | 1.3 | 1.3 | 2.5 | 1.9 |
| TRINITY_DN12632_c0_g1 | Calcium-binding protein CML | 4.4 | 2.5 | 1.8 | 1.8 |
| TRINITY_DN9511_c0_g1 | Calcium-binding protein CML | 4.6 | 2.3 | 1.8 | 2.5 |
| TRINITY_DN19461_c0_g1 | Calcium-binding protein CML | 2.1 | 0.8 | 8.2 | 2.6 |
| TRINITY_DN3764_c0_g1 | Calcium-binding protein CML | 1.8 | 1.8 | 1.7 | 2.0 |
| TRINITY_DN2202_c1_g1 | Mitogen-activated protein kinase kinase 2 | 2.1 | 1.4 | 1.7 | 1.6 |
| TRINITY_DN5338_c2_g1 | Mitogen-activated protein kinase kinase 9 | 2.6 | 2.0 | 2.4 | 1.4 |
| TRINITY_DN14276_c0_g1 | Mitogen-activated protein kinase kinase kinase 17 | 1.0 | 1.2 | 2.0 | 8.1 |
| TRINITY_DN4010_c3_g1 | Mitogen-activated protein kinase kinase kinase 17 | 1.7 | 0.8 | 3.1 | 2.4 |
| TRINITY_DN18119_c0_g1 | Transcription factor TGA | 1.7 | 2.2 | 1.8 | 2.4 |
| TRINITY_DN6680_c0_g1 | Pathogenesis-related protein 1 | 8.0 | 3.0 | 21.0 | 43.2 |
| TRINITY_DN7113_c0_g1 | Pathogenesis-related protein 1 | Inf | Inf | 161.7 | 18.4 |
| TRINITY_DN65370_c0_g1 | Pathogenesis-related protein 1 | Inf | Inf | Inf | Inf |
| TRINITY_DN35589_c0_g1 | Pathogenesis-related protein 1 | Inf | Inf | 57.3 | 11.0 |
| TRINITY_DN1033_c0_g1 | Respiratory burst oxidase | 2.2 | 2.0 | 4.3 | 4.6 |
| TRINITY_DN10719_c0_g1 | Respiratory burst oxidase | 3.2 | 2.2 | 9.3 | 4.7 |
| TRINITY_DN26735_c0_g1 | Respiratory burst oxidase | 2.4 | 2.0 | 10.9 | 18.9 |
| TRINITY_DN7466_c0_g1 | Respiratory burst oxidase | 2.4 | 2.2 | 3.8 | 2.5 |
| TRINITY_DN22579_c0_g1 | Respiratory burst oxidase | 3.1 | 2.2 | 2.8 | 1.0 |
| TRINITY_DN4896_c0_g2 | Respiratory burst oxidase | 3.0 | 1.4 | 2.2 | 1.2 |
| TRINITY_DN11963_c0_g1 | Peroxidase | 8.0 | 3.7 | 17.8 | 7.5 |
| TRINITY_DN22971_c0_g1 | Peroxidase | 8.8 | 3.8 | 107.3 | 18.6 |
| TRINITY_DN3139_c0_g1 | Peroxidase | 1.1 | 2.2 | 9.9 | 7.8 |
| TRINITY_DN3355_c0_g1 | Peroxidase | 107.9 | 44.1 | 45.5 | 18.9 |
| TRINITY_DN3926_c1_g1 | Peroxidase | 3.3 | 2.1 | 2.5 | 1.9 |
| TRINITY_DN427_c0_g1 | Peroxidase | 2.5 | 3.4 | 7.8 | 8.6 |
| TRINITY_DN4768_c1_g1 | Peroxidase | 0.1 | 0.6 | 32.6 | 10.0 |
| TRINITY_DN4876_c1_g1 | Peroxidase | 4.2 | 2.6 | 15.5 | 10.2 |
| TRINITY_DN4981_c1_g1 | Peroxidase | 1.6 | 1.7 | 3.9 | 3.8 |
| TRINITY_DN5925_c1_g1 | Peroxidase | 165.8 | 11.7 | 564.2 | 745.9 |
| TRINITY_DN7911_c0_g1 | Peroxidase | 2.2 | 1.7 | 1.9 | 2.5 |
| TRINITY_DN2677_c1_g1 | Polyphenol oxidase | 2.6 | 1.8 | 7.8 | 10.6 |
| TRINITY_DN15244_c0_g1 | Catalase | 2.7 | 2.4 | 2.5 | 2.0 |
| TRINITY_DN66501_c0_g1 | Catalase | 2.2 | 2.1 | 2.5 | 2.2 |
| TRINITY_DN3951_c11_g2 | Catalase | 2.9 | 1.9 | 3.1 | 2.5 |
| TRINITY_DN25766_c1_g1 | Catalase | 1.8 | 2.3 | 3.4 | 3.3 |

**Table S5. Detected metabolites using widely targeted metabolome analyses in Resistant and susceptible *Cyclocarya paliurus* leaves in response to *Colletotrichum fructicola* infection.**

Note: Large table that provides by independent file.

| **Table S6. Geographical and climatical information of the six cultivars of *Cyclocarya paliurus* used in this study.** | | | | | |
| --- | --- | --- | --- | --- | --- |
| Natural cultivars | Province | Latitude (N) | Longitude (E) | Altitude (m) | Annual mean temperature (°C) |
| Anji | Zhejiang | 30°24'36" | 119°38'24" | 540 | 15.4 |
| Jinggangshan | Jiangxi | 26°30'36" | 114°6'36" | 967 | 13.8 |
| Jinzhongshan | Guangxi | 24°36'36" | 104°57'00" | 1798 | 16.2 |
| Lipin | Guizhou | 26°20'24" | 109°14'24" | 727 | 15.6 |
| Muchuan | Sichuan | 28°58'00" | 103°47'00" | 1200 | 15.7 |
| Wufeng | Hubei | 30°17'00" | 110°80'00" | 969 | 16.7 |

| **Table S7. Primers used for the quantification of gene expression levels**. | | |
| --- | --- | --- |
| Gene ID/name | Functional annotation | Primer sequence (forward/reverse 5' to 3') |
| TRINITY_DN1033_c0_g1 | Respiratory burst oxidase homolog (*RBOH*) | AACTTAAGGGTCTCAGCCGC |
|  |  | AAGGCCCAATACGAAGGTGG |
| TRINITY_DN15244_c0_g1 | Catalase (*CAT*) | GACCCTCGAGGTTTTGCTGT |
|  |  | AGGAAGTCGAGAACCCTCCA |
| TRINITY_DN7911_c0_g1 | Peroxidase (*POD*) | CCCATCTTCAGCATTCCCGT |
|  |  | CCAGAACGTCACAGGAGACC |
| TRINITY_DN5694_c0_g1 | LRR receptor-like serine/threonine-protein kinase FLS1 (*RLK*) | ACCGGAGAAATGAGCTTGAAGT |
|  |  | AGGTGAGTCGGCACAACAAT |
| TRINITY_DN501_c1_g2 | Phenylalanine ammonia-lyase (*PAL*) | TGGCAATGGCCTCCATGATT |
|  |  | TCACCACAGGTTTTGGTGCT |
| TRINITY_DN4977_c0_g1 | Chalcone isomerase (*CHI*) | CTCACTCACCAGTACCGTGG |
|  |  | ACTCACACATGCCAGTCAGG |
| TRINITY_DN10902_c0_g1 | Flavonol synthase (*FLS*) | CATTTCTTGGCTCCTCCTTCT |
|  |  | TCCAGCAAGGTGTCTGTTATC |
| TRINITY_DN1003_c0_g2 | Leucoanthocyanidin reductase (*LAR*) | GCCACACTCAAATCCCTCCA |
|  |  | GGTCCGCTCTGTCTATGTCG |
| *Cp18sRNA* | The endogenous reference gene for *Cyclocarya paliurus* | AGTATGGTCGCAAGGCTGAAA |
|  |  | CAGACAAATCGCTCCACCAA |
| CGMCC3_g6100 | Laccase (*LAC*) | CGTCCGAAGTTGCTCTTCCT |
|  |  | TGGGGTATCCTGCTTGGGTA |
| CGMCC3_g6455 | Glutamate dehydrogenase (*Gdh*) | AGTTCGAGCAGGCCTACAAC |
|  |  | GACAGGTTGACGGTAGGGTG |
| CGMCC3_g13653 | C2H2 transcription factor (*C_2_H_2_*) | CTCCTCGCGATCACTCTCAC |
|  |  | GAAGGTGGCAGGATGCTTCT |
| CGMCC3_g5684 | Endocytosis (*End*) | TCAAGTTCTTCCGCGGTCTC |
|  |  | TCGACGAGCTTGTTGAGCTT |
| *CfβTUB* | The endogenous reference gene for *Colletotrichum fructicola* | CCACTTCCCTTTGGTCGCTTAC |
|  |  | CATGGTCATCTCCTGGACAGAGT |
